# Impact of the COVID-19 Pandemic on BMI and Obesity among Underrepresented Populations: A Longitudinal Analysis of the All of Us Dataset

**DOI:** 10.21203/rs.3.rs-7567825/v1

**Published:** 2025-10-17

**Authors:** Abdul-Hanan Saani Inusah, Huiyi Xia, Atena Pasha, Zhenlong Li, Andrew T Kaczynski, Xiaoming Li, Shan Qiao

**Affiliations:** University of South Carolina; University of South Carolina; University of South Carolina; The Pennsylvania State University; University of South Carolina; University of South Carolina; University of South Carolina

**Keywords:** Body mass index (BMI), obesity, COVID-19 pandemic, sociodemographic disparities, pandemic preparedness, health disparities

## Abstract

**Introduction:**

The COVID-19 pandemic and its prevention measures (e.g., quarantine, social distancing, and shutdown) significantly affected people’s physical activity and lifestyle, potentially increasing Body Mass Index (BMI) and risk of obesity. This study provides a comprehensive analysis to examine changes in BMI and obesity rate across pre-COVID, COVID, and post-COVID periods, and to identify the sociodemographic correlates of BMI and obesity change.

**Methods:**

Longitudinal electronic health record data from the All of Us Research Program for adults ≥ 18 years with at least one BMI record in each period (N = 38,632) were analyzed. Periods were defined as pre-COVID (Jan 1, 2018–Mar 12, 2020), COVID (Mar 13, 2020–Dec 31, 2021), and post-COVID (Jan 1, 2022–Oct 31, 2023). We modeled BMI with a linear mixed-effects model and obesity (BMI ≥ 30) with a GEE logit model, adjusting for age, sex, race, ethnicity, income, and employment; time was modeled with linear and quadratic terms, and we tested time × subgroup interactions.

**Results:**

Mean BMI increased from 30.05 pre-COVID to 30.14 during COVID, then declined to 29.96 post-COVID; obesity prevalence followed a similar pattern (43.5% → 44.1% → 43.2%). In adjusted models, linear time effects were positive and the quadratic terms negative for both outcomes, indicating non-linear (increase-then-partial-reversal) trends (BMI: β_time = 0.216; β_time^2^ = −0.018; obesity: aOR_time = 1.052; aOR_time^2^ = 0.996; all p < 0.001). Female, Black and Hispanic/Latino participants had higher BMI and greater odds of obesity than their counterparts, while higher income showed a protective association. Significant time × subgroup interactions were observed, with age and employment status showed the most consistent effects across both outcomes.

**Conclusion:**

BMI and obesity increased during the pandemic and partially reversed afterward, but persistent disparities remained, with higher risk among women, Black and Hispanic/Latino adults, and lower-income groups. Results highlight the impact of COVID-19 on obesity risk and emphasize the need for proactive measures to promote healthier lifestyle and weight management strategies for vulnerable groups in future public health preparedness plans.

## Introduction

The COVID-19 pandemic disrupted daily routines worldwide, leading to significant changes in health-related behaviors. Lockdowns, remote schooling/working, and travel restrictions limited access to physical activity opportunities and changed dietary patterns across all age groups (1, 2). These behavioral disruptions, along with increased stress and social isolation, created an environment that contributed to weight gain and worsened the existing obesity epidemic (1, 3, 4). For underserved populations, this closure also reduced resources that often provide access to meals and emotional support (5, 6). These public health measures, though necessary for curbing viral spread, had unintended consequences on population-level BMI trends and metabolic health, including elevated rates of obesity (7, 8).

Obesity was a global public health concern even before the pandemic. In the U.S., more than 42% of adults were classified as obese in 2017–2018, and these rates would continue to rise in the following years (9, 10). The COVID-19 pandemic further exacerbated these trends as studies have shown significant increases in average BMI and obesity rates during the pandemic, with both adults and children experiencing faster weight gain compared to pre-pandemic periods (8, 11, 12). In this regard, longitudinal analyses revealed average monthly weight gains of 1–1.5 pounds during shelter-in-place mandates (13, 14).

However, the impact of these changes has not been distributed evenly, as racial and ethnic minority groups and individuals with lower socioeconomic status experienced a disproportionate burden of both COVID-19 outcomes and obesity-related risk factors (15, 16). These disparities reflect long-standing structural inequities in access to healthcare, healthy foods, safe neighborhoods, and economic stability. For instance, Black and Hispanic populations, already more likely to be obese, also faced greater job insecurity, income loss, and limited access to recreational spaces during the pandemic (17, 18). Simultaneously, these groups experienced higher rates of COVID-19 morbidity and mortality, further compounding their health risks (19, 20). These socioeconomic stressors, psychological distress, and food insecurity are all established correlates of increased BMI (21–23).

Despite growing literature on pandemic-related weight changes, existing studies often suffer from key limitations that restrict their policy relevance and scientific generalizability. Many have focused narrowly on comparing only two time points (e.g., pre- versus during-pandemic), without examining how weight trends evolved after public health restrictions were lifted (8, 24). Others have relied on convenience samples, geographically limited datasets, or clinical cohorts, making it difficult to draw conclusions about national trends or diverse populations (11, 12). Moreover, prior studies often lacked detailed sociodemographic factors (e.g., sex, employment status, income, race/ethnicity) that are critical to understanding disparities in obesity outcomes. This gap is critical, given that the pandemic may have differentially affected health behaviors and obesity risk among historically marginalized groups.

To address these limitations, this study aims to provide a comprehensive longitudinal analysis of BMI and obesity trends across pre-pandemic, during-pandemic, and post-pandemic periods using the National Institutes of Health All of Us Program, and to identify sociodemographic factors, such as sex, race, ethnicity, income, and employment status, that contribute to differences in obesity prevalence over time. Focusing on vulnerable and historically underrepresented groups, this analysis offers important insight into the long-term health consequences of the COVID-19 pandemic.

## Methods

### Data Source and Participants

We conducted a retrospective cohort study using data from the All of Us (AoU) Program (Curated Data Repository Version 8, Controlled Tier). The AoU program is a nationwide precision medicine initiative that collects diverse health data from at least one million participants across the United States (25). This dataset includes electronic health record (EHR) and survey information for a broad, geographically and demographically diverse cohort. Approximately 78% of participants belong to groups historically underrepresented in biomedical research, including racial and ethnic minorities, individuals with lower income and educational attainment, and sexual and gender minorities (26). EHR data in AoU are collected by participating health care provider organizations and submitted to the program’s Data and Research Center, which harmonizes and curates the data for research use (25). Prior validation studies have confirmed the reliability of these data, including replication of known clinical patterns and treatment pathways, thereby supporting the dataset’s quality and utility for population health research (26).

For this study, we utilized deidentified data from the Controlled Tier of AoU. All adults aged 18 years or older with available BMI measurements in each of the three pandemic-related time periods were eligible for inclusion. These periods were defined as: pre-COVID (January 1, 2018-March 12, 2020), COVID (March 13, 2020-December 31, 2021), and post-COVID (January 1, 2022-October 31, 2023). Although the worldwide COVID-19 pandemic did not end officially until May 2023 (27), we defined the post-COVID period as beginning January 1, 2022 based on behavioral and contextual changes most relevant to obesity research. By late 2021, many restrictions had eased, in-person activities had resumed, and population-level vaccination and immunity had increased, signaling a shift in daily routines, physical activity patterns, and food environments (28). These factors justified our decision to treat 2022 onward as a distinct post-pandemic phase aligned with the return to more typical lifestyle conditions

To reduce the influence of extreme age outliers, we excluded participants with age outside the 2nd to 97th percentile (n = 97). A final analytic sample of 38,632 participants remained after applying the inclusion and exclusion criteria.

### Outcome Measures

#### Outcome Measures

Two outcome measures were examined: BMI as a continuous variable and obesity as a binary variable. BMI was defined as weight in kilograms divided by height in meters squared, and BMI values were obtained from AoU-calculated EHR data fields. For participants with multiple BMI measurements within a single calendar year, all BMI values for that year were averaged to produce one representative annual BMI value per person. This annual average was used to reduce intra-individual measurement variability and provide a stable estimate of BMI for each calendar year. Using these annual BMI values, we defined obesity status as a binary indicator (obese vs. non-obese). Obesity was classified as an average BMI ≥ 30 kg/m^2^ for the year, consistent with standard clinical definitions (29). Each participant therefore had a BMI value and corresponding obesity classification for each of the three time periods. For 2020, BMI values prior to March 13 were assigned to the pre-COVID period and those on or after March 13 were assigned to the COVID period, ensuring that annual averages did not combine measurements across phases.

### Covariates

Sociodemographic covariates included age, sex, race, ethnicity, household income, and employment status. Age was treated as a continuous variable (it was also categorized into younger (≤ 50) vs. older (> 50) adult groups for descriptive analyses). Sex was categorized as male or female. Race was classified as White, Black or African American, and Asian/Other/Unknown, while ethnicity was categorized as Hispanic or Latino/Unknown versus non-Hispanic. Annual household income was grouped into four categories: less than $25,000, $25,000–$50,000, $50,000–$100,000, and over $100,000. Employment status was categorized as employed versus unemployed. These variables were included as covariates in the regression models to control for potential confounding.

### Statistical Analysis

We conducted descriptive analyses to examine the trends of BMI and obesity in the whole sample and by demographic subgroups. The overall differences between subgroups by each demographic variable were tested using repeated-measures ANOVA (for BMI) and bivariate generalized estimating equation (GEE) models (for obesity). We then employed multivariable regression models to assess changes in BMI and obesity across the three time periods. A linear mixed-effects model was used for BMI (continuous outcome), and a GEE with a logit link was used for obesity (binary outcome). Time was modeled as both categorical (pre-COVID [reference], COVID, post-COVID) and continuous with a quadratic term to capture potential non-linear trends.

All models adjusted for age, sex, race, ethnicity, household income level, and employment status. Two-way interaction terms between time and each sociodemographic factor were included to test for subgroup differences over time. The mixed-effects model incorporated a person-specific random intercept to account for repeated measures within individuals, while the GEE used an exchangeable working correlation structure to account for within-person correlation.

Results from the mixed-effects model are reported as β coefficients (adjusted mean differences) with 95% confidence intervals, and results from the GEE are reported as adjusted odds ratios (aORs) with 95% confidence intervals. Two-sided p-values < 0.05 were considered statistically significant. All analyses were conducted using R software (version 4.2.0).

### Ethics

This study was conducted using de-identified data from the AoU Research Program, which operates under a central institutional review board (IRB) approval. All participants in AoU provided informed consent at enrollment. As this study used de-identified secondary data, no additional IRB approval was required.

## Results

### Sample Characteristics.

This analysis includes 38,632 adults aged 18 years and older with at least one BMI record during each of the three designated time periods: pre-COVID, COVID, and post-COVID. As shown in Table 1, the mean age at baseline was 50.6 years (SD = 13.7). The majority of participants were female (66.8%), White (59.7%), and non-Hispanic (81.6%). Nearly half of the sample reported household incomes over $100,000, while 54% were employed and 46% were unemployed.

### BMI and Obesity Prevalence Trends

As shown in Table 1, mean BMI increased from 30.05 (SD = 0.015) in the pre-COVID period to 30.14 (SD = 0.114) during COVID, then declined to 29.96 (SD = 0.015) in the post-COVID period. Obesity prevalence followed a similar pattern, rising from 43.5% (SD = 0.122) pre-COVID to 44.1% (SD = 0.845) during COVID, and then decreasing to 43.2% (SD = 0.090) in the post-COVID period. These results indicate that both BMI and obesity rates increased during the pandemic but partially reversed afterward, returning close to pre-pandemic levels.

All subgroup comparisons for BMI were statistically significant (p < 0.001). For example, younger adults, females, and participants with lower income consistently showed higher BMI. Obesity trends were significant for all other sociodemographic groups except race, ethnicity or income groups (Table 1).

### Multivariate Analysis (BMI)

#### Main Effects of Demographic Factors

As shown in Table 2, older adults (age ≥ 50) had significantly lower BMI than younger adults (β = − 0.477, p < 0.001). Female participants had a higher BMI than males (β = 0.621, *p* < 0.001). Black/African American participants had higher BMI compared to White participants (β = 2.861, *p* < 0.001), whereas Asian/Other participants did not differ significantly (β = − 0.030, *p* = 0.815). Hispanic/Latino ethnicity was associated with higher BMI than non-Hispanic ethnicity (β = 0.699, *p* < 0.001). Income was inversely related to BMI: participants earning >$100,000 had significantly lower BMI than those earning <$25,000 (β = − 2.188, *p* < 0.001), and those with $50,000–100,000 also had lower BMI (β = − 0.842, *p* < 0.001). In contrast, the $25,000–50,000 group did not differ significantly from the lowest income group (β = 0.074, *p* = 0.553). Employment status was not significantly associated with BMI (β = 0.094, p = 0.199).

#### Time Effects

BMI increased from the pre- to COVID period (β = 0.216, *p* < 0.001). However, the quadratic term was negative (β = − 0.018, *p* < 0.001), indicating a non-linear trend, with BMI rising during the COVID period but then slowing and slightly reversing by the post-COVID period (Table 2).

#### Interaction Effects

As shown in Table 2, differences in BMI changes over time were observed across several demographic groups. There was a significant age × time interaction (*p* < 0.001), with older adults showing smaller changes in BMI compared to younger adults (β = − 0.163, *p* < 0.001). A sex × time interaction indicated that females had slightly greater changes in BMI over time than males (β = 0.017, *p* = 0.013). Black participants showed smaller changes in BMI over time compared to White participants (β = − 0.042, *p* < 0.001), whereas Asian/Other participants did not differ significantly (β = − 0.001, *p* = 0.936). Hispanic participants exhibited smaller changes in BMI than non-Hispanics (β = − 0.026, *p* = 0.049). Unemployed participants also had smaller changes in BMI compared to employed participants (β = − 0.055, *p* < 0.001). No significant time interactions were observed for income groups.

### Multivariate Analysis (Obesity)

#### Main Effects of Demographic Factors

As shown in Table 3, older adults (≥ 50 years) had lower odds of obesity compared to younger adults (aOR = 0.906, *p* < 0.001). Female participants had higher odds of obesity than male participants (aOR = 1.258, *p* < 0.001). Black/African American participants had about two-fold higher odds of obesity compared to White participants (aOR = 2.052, *p* < 0.001), while Asian/Other participants did not differ significantly from White participants (aOR = 1.023, *p* = 0.544). Hispanic/Latino ethnicity was associated with higher odds of obesity compared to non-Hispanic ethnicity (aOR = 1.263, *p* < 0.001). Income showed a clear gradient: individuals earning >$100,000 had substantially lower odds of obesity compared to those earning <$25,000 (aOR = 0.571, *p* < 0.001), and those earning $50,000–100,000 also had lower odds (aOR = 0.817, *p* < 0.001). The $25,000–50,000 group had slightly higher odds of obesity compared to the lowest income group, though this effect was small (aOR = 1.074, *p* = 0.045). There was no statistically significant association between employment status and obesity (aOR = 1.032, *p* = 0.174).

#### Time Effects

As shown in Table 3, the odds of obesity increased across the study periods (aOR per period = 1.052, *p* < 0.001). The quadratic (aOR = 0.996, *p* < 0.001) indicated a non-linear trend, with obesity odds increasing during the COVID period but then leveling off or slightly declining by the post-COVID period.

#### Interaction Effects

As shown in Table 3, differences in changes in obesity odds over time were observed across some demographic groups. There was a significant age × time interaction, with older adults showing smaller changes in obesity odds compared to younger adults, indicating that obesity odds increased more among younger adults while remaining relatively stable in the older group (aOR = 0.960, *p* < 0.001). Similarly, unemployed participants also had reduced changes in obesity odds compared to employed participants, suggesting that obesity odds rose more among employed individuals, while remaining more stable among those who were unemployed (aOR = 0.986, *p* < 0.001). No significant time interactions were observed for sex, race, or income groups.

## Discussion

Our analysis revealed significant temporal changes in BMI and obesity across the three pandemic-related periods, alongside persistent sociodemographic disparities. Both BMI and obesity increased during the COVID-19 period compared to the pre-pandemic baseline. In the post-COVID period, both outcomes showed non-linear changes, with BMI and obesity odds either plateauing or reversing slightly. These findings highlight the pandemic period as a turning point, characterized by weight gain and rising obesity, followed by a shift toward stabilization and partial reversal in the post-COVID period.

Persistent disparities in BMI and obesity were evident across sociodemographic groups. Women, Black and Hispanic participants, and those with lower incomes consistently had higher BMI and greater odds of obesity compared to their counterparts throughout the pre-COVID, COVID, and post-COVID periods. These inequalities remained evident despite shifts in overall trends. However, changes over time were not uniform across all groups. Interaction effects were most consistent for age and employment status, with older adults and unemployed participants experiencing smaller changes in BMI and obesity over time compared to their younger and employed counterparts.

The observed increase in BMI during the COVID-19 period is consistent with prior evidence linking pandemic-related disruptions to weight gain (30). Lockdowns, social distancing, and prolonged home confinement were associated with reduced physical activity, greater sedentary behavior, and shifts toward more energy-dense diets (16, 31). Psychological stress and anxiety may also have contributed to elevated food intake, with stress-induced hormonal changes, such as increased cortisol, potentially enhancing appetite and cravings for high-calorie foods (16, 31). These mechanisms likely contributed to the population-level rise in BMI and obesity observed in our study. In the post-pandemic period, we found that mean BMI and obesity showed declines, which may reflect the partial normalization of health behaviors as public health restrictions eased. Some survey-based studies have reported that changes in exercise and diet during lockdown were temporary and tended to reverse after restrictions were lifted (32).

Our findings reaffirm entrenched disparities in BMI and obesity risk among Black, Hispanic, female, unemployed, and low-income participants. These disparities are not new; rather, the COVID-19 pandemic appears to have exacerbated longstanding inequities. Prior to the pandemic, national data already showed that obesity disproportionately affected non-Hispanic Black (49.6%) and Hispanic (44.8%) adults (33). Non-Hispanic Black women, in particular, faced the highest obesity prevalence (56.9%) of any major demographic group (33), reflecting the intersection of racial and gender-based disadvantage. These disparities are deeply rooted in structural and social inequities. Black and Hispanic communities in the U.S. have historically faced restricted access to the social determinants of health that support healthy weight, such as economic stability, affordable nutritious food, and safe environments for physical activity (34–36). Neighborhood segregation, chronic stress exposure, food deserts, and underinvestment in health infrastructure contribute to these persistent gaps (37, 38).

The pandemic intensified these vulnerabilities. As lockdowns disrupted food supply chains and shut down schools, income sources, and public services, food insecurity sharply increased, particularly among low-income households and communities of color (16). Essential food access systems were strained, deepening pre-existing nutritional inequities and limiting options for healthy eating. Moreover, lower-income individuals experienced greater disruptions in physical activity and access to care due to transportation barriers, gym closures, and heightened fear of infection (39–41). Meanwhile, higher-income groups were more likely to preserve access to exercise spaces and healthy diets, widening behavioral and health disparities (32, 42).

The rise in BMI and obesity during the COVID-19 pandemic offers crucial lessons for public health preparedness and response. Future public health emergencies must incorporate strategies that safeguard nutritional health and physical activity. First, emergency response plans must include measures to maintain access to healthy, affordable food (43). Governments should ensure the continuity of community nutrition programs (e.g., Supplemental Nutrition Assistance Program [SNAP], food banks, school meal services), provide transportation support, and adopt contingency plans for mobile food distribution in high-risk areas and groups. Second, physical activity promotion should be an integral component of pandemic preparedness (44). Public health agencies must provide accessible resources for maintaining safe physical activity, including guidance on at-home exercises and socially distanced routines. Policies that keep parks and outdoor recreational spaces open safely should be prioritized. Additionally, leveraging digital health platforms to disseminate culturally tailored fitness content can help maintain physical activity among diverse communities (44). Third, culturally and contextually relevant communication is essential for high-risk populations (42). Interventions must be co-developed with trusted local organizations and leaders who can disseminate accurate, culturally grounded information. For example, community health workers serving Black and Hispanic neighborhoods can deliver targeted health messaging, stress-reduction strategies, and nutrition education in relevant languages and formats (45). Fourth, the integration of mental health support into emergency response frameworks is critical (46). Telehealth platforms should be used to provide virtual counseling and nutritional guidance early in a public health emergency to mitigate downstream health risks (47).

This study has a number of limitations that warrant consideration. First, although the AoU dataset offers rich longitudinal health data with strong representation from historically underrepresented populations, it is not fully representative of the general U.S. population. As such, findings should be interpreted with caution when generalizing to the broader population. Second, obesity was assessed solely by using BMI, which, while widely used, does not account for body composition. Future studies should incorporate complementary measures such as waist circumference and body fat percentage to more accurately capture adiposity. Third, although some participants had multiple BMI records, others had only a single or limited number of observations. While we used average values for participants with multiple measurements, findings should be interpreted carefully due to potential variability in measurement frequency. Finally, although the AoU dataset includes information on other potential predictors of weight change, such as physical activity and stress and coping strategies during the pandemic based on its Fitbit and survey data, these variables were not included in the present analysis due to the scope of our study. Future studies should leverage these behavioral and psychosocial data to more comprehensively examine factors driving BMI and obesity changes during the COVID-19 pandemic.

## Conclusions

This study highlights the lasting impact of the COVID-19 pandemic on BMI and obesity, with increases observed during the pandemic followed by partial reversal in the post-pandemic period. Persistent disparities across sex, race, ethnicity, income, and employment emphasize the disproportionate burden among historically marginalized populations. These findings emphasize the urgency of long-term, sustained efforts to reduce obesity prevalence and eliminate inequities. The post-pandemic period presents a critical window of opportunity to invest in long-term prevention, build systemic resilience, and narrow the deeply entrenched gaps in obesity burden.

## Figures and Tables

**Table 1:** Sociodemographic Characteristics and Descriptive Trends in BMI and Obesity across Pre-COVID, COVID-19, and Post-COVID Periods (N = 38,632)

Variables	Overall	BMI: Mean (SD)				Obesity Prevalence: n (%) or % (SE)			
	(N = 38632)	PreCOVID	COVID	Post-COVID	P-value	Pre-COVID	COVID	Post-COVID	P-value
					(ANOVA)				(GEE)
Total sample		30.047(0.015)	30.137 (0.114)	29.964 (0.015)	n/a	43.465 (SD = 0.122)	44.128 (SD = 0.845)	43.227 (SD = 0.090)	n/a
Overall mean Age (SD)	50.6 (13.7)								
Median [Min, Max]	52.0 [21.0, 76.0]								
*Age subcategories*									
Younger adults (< 50 years)	17354(44.9%)	30.61(7.15)	30.86(7.24)	30.86(7.15)	0.0004	8115(46.8)	8366(48.2)	8411(48.5)	
Older adults (≥ 50 years)	21278(55.1%)	29.52(6.14)	29.37(6.19)	29.15(6.11)		8551(40.2)	8399(39.5)	8113(38.1)	< 0.001
*Sex*									
Male	12844(33.2%)	29.38(5.58)	29.3(5.65)	29.19(5.61)	< 0.001	4913(38.3)	4862(37.9)	4745(36.9)	
Female	25788(66.8%)	30.32(7.09)	30.41(7.17)	30.29(7.09)		11753(45.6)	11903(46.2)	11779(45.7)	< 0.001
*Race*									
White	23044(59.7%)	29.24(6.39)	29.24(6.46)	29.18(6.39)	< 0.001	8762(38.0)	8747(38.0)	8717(37.8)	
Black/African American	6641(17.2%)	32.67(7.27)	32.74(7.34)	32.47(7.32)		3950(59.5)	3997(60.2)	3893(58.6)	0.060
Asian/Other/Unknown	8947(23.2%)	30.01(6.26)	30.11(6.36)	29.94(6.3)		3954(44.2)	4021(44.9)	3914(43.7)	0.640
*Ethnicity*									
NonHispanic/Latino	31524(81.6%)	29.95(6.76)	29.98(6.84)	29.87(6.77)	< 0.001	13415(42.6)	13431(42.6)	13302(42.2)	
Hispanic/Latino	7108(18.4%)	30.24(6.08)	30.32(6.14)	30.15(6.11)		3251(45.7)	3334(46.9)	3222(45.3)	0.830
*Annual Income Level (USD)*									
< 25k	7487(19.4%)	31.83(7.22)	31.98(7.31)	31.69(7.28)	< 0.001	4078(54.5)	4141(55.3)	4035(53.9)	
25k - 50k	4867(12.6%)	31.39(6.97)	31.46(6.98)	31.32(6.96)		2576(52.9)	2591(53.2)	2546(52.3)	0.583
50k - 100k	8000(20.7%)	30.03(6.52)	30.01(6.6)	29.96(6.56)		3470(43.3)	3453(43.2)	3444(43.0)	0.377
>100k	18278(47.3%)	28.88(6.09)	28.88(6.18)	28.81(6.08)		6542(35.8)	6580(36.0)	6499(35.6)	0.287
*Employment Status*									
Employed	17619(45.6%)	29.82(6.61)	29.95(6.71)	29.97(6.64)	0.0002	7370(41.8)	7492(42.5)	7539(42.8)	
Unemployed	21013(54.4%)	30.16(6.66)	30.12(6.73)	29.88(6.66)		9296(44.2)	9273(44.1)	8985(42.8)	< 0.001

Values are presented as mean (SD) for BMI and count (percentage) for obesity prevalence. P-values represent overall differences between subgroups within each variable, based on repeated-measures ANOVA (BMI) and bivariate GEE models (obesity).

**Table 2:** Mixed-Effects Regression Results for BMI with Main Effects and Time × Subgroup Interactions across Pre-COVID, COVID-19, and Post-COVID Periods (N = 38,632)

Variables	Coefficients	P value	95CI
*Age*			
Younger adults (< 50 years)	*reference*		
Older adults (≥ 50 years)	−0.477	0.000	(−0.626, −0.328)
*Sex*			
Male	*reference*		
Female	0.621	0.000	(0.481, 0.761)
*Race*			
White	*reference*		
Black/African American	2.861	0.000	(2.673, 3.049)
Asian/Other/Unknown	0.030	0.815	(−0.22, 0.279)
*Ethnicity*			
Non-Hispanic/Latino	*reference*		
Hispanic/Latino	0.699	0.000	(0.432, 0.966)
*Annual Income Level (USD)*			
<25k	*reference*		
25k-50k	0.074	0.550	(−0.168, 0.315)
50k-100k	−0.875	0.000	(−1.096, −0.654)
>100k	−2.168	0.000	(−2.356, −1.980)
*Employment Status*			
Employed	*reference*		
Unemployed	0.094	0.199	(−0.049, 0.238)
Time	0.216	0.000	(0.190, 0.242)
Time * Time	−0.018	0.000	(−0.021, −0.016)
Demo * Time Interaction Age * Time			
Older adults * Time	−0.163	0.000	(−0.178, −0.148)
Sex * Time Female * Time Race * Time	0.017	0.013	(0.004, 0.031)
Black/African American * Time	−0.042	0.000	(−0.060, −0.024)
Asian/Other * Time Ethnicity *Time	−0.001	0.936	(−0.025, 0.023)
Hispanic/Latino * Time	−0.026	0.049	(−0.052, 0.000)
Employment status * Time Unemployed * Time	−0.058	0.000	(−0.072, −0.044)
Income * Time			
25k-50k × Time	−0.005	0.703	(−0.028,0.019)
50k-100k × Time	−0.002	0.864	(−0.023,0.020)
> 100k × Time	−0.006	0.546	(−0.024,0.013)

**Table 3:** Generalized Estimating Equation (GEE) Models of Obesity: Main Effects and Time Interactions Across Pre-, During-, and Post-COVID Periods (N = 38,632)

Variables	Estimate	aOR	95CI	P Value
*Age*				
Younger adults (< 50 years)	*reference*			
Older adults (≥ 50 years)	−0.099	0.906	(0.865,0.949)	0.000
*Sex*				
Male	*reference*			
Female	0.229	1.258	(1.203,1.315)	0.000
*Race*				
White	*reference*			
Black/African American	0.719	2.052	(1.944,2.166)	0.000
Asian/Other/Unknown	0.023	1.023	(0.951,1.100)	0.544
*Ethnicity*				
Non-Hispanic/Latino	*reference*			
Hispanic/Latino	0.234	1.263	(1.166,1.368)	0.000
*Annual Income Level (USD)*				
< 25k	*reference*			
25k- 50k	0.071	1.074	(1.002,1.151)	0.045
50k -100k	−0.202	0.817	(0.767,0.871)	0.000
> 100k	−0.561	0.571	(0.540,0.603)	0.000
*Employment Status*				
Employed	*reference*			
Unemployed	0.031	1.032	(0.986,1.079)	0.174
Time	0.050	1.052	(1.039,1.063)	0.000
Time * Time	−0.004	0.996	(0.994,0.997)	0.000
Demo * Time Interaction Age * Time				
Older adults * Time Employment * Time	−0.041	0.960	(0.953,0.967)	0.000
Unemployed * Time	−0.015	0.986	(0.979,0.992)	0.000

No significant time interactions were observed for sex, race, ethnicity or income groups (p-value > 0.05)

## Data Availability

The data used in this study are available through the National Institutes of Health All of Us Research Program

## Abbreviations

AoU: All of Us Research Program
BMI: Body Mass Index
COVID: 19–Coronavirus Disease 2019
EHR: Electronic Health Record
GEE: Generalized Estimating Equations
IRB: Institutional Review Board
SD: Standard Deviation
SNAP: Supplemental Nutrition Assistance Program
aOR: Adjusted Odds Ratio
β(Beta coefficient): Regression Coefficient
WHO: World Health Organization
